# *FlexiS*—A Flexible Sensor Node Platform for the Internet of Things

**DOI:** 10.3390/s21155154

**Published:** 2021-07-29

**Authors:** Duc Minh Pham, Syed Mahfuzul Aziz

**Affiliations:** UniSA STEM, University of South Australia, Mawson Lakes, SA 5095, Australia; duc.pham@unisa.edu.au

**Keywords:** Internet of Things (IoT), wireless sensor networks (WSN), sensor node, field-programmable gate array (FPGA), FPGA programming, energy efficiency

## Abstract

In recent years, significant research and development efforts have been made to transform the Internet of Things (IoT) from a futuristic vision to reality. The IoT is expected to deliver huge economic benefits through improved infrastructure and productivity in almost all sectors. At the core of the IoT are the distributed sensing devices or sensor nodes that collect and communicate information about physical entities in the environment. These sensing platforms have traditionally been developed around off-the-shelf microcontrollers. Field-Programmable Gate Arrays (FPGA) have been used in some of the recent sensor nodes due to their inherent flexibility and high processing capability. FPGAs can be exploited to huge advantage because the sensor nodes can be configured to adapt their functionality and performance to changing requirements. In this paper, *FlexiS*, a high performance and flexible sensor node platform based on FPGA, is presented. Test results show that *FlexiS* is suitable for data and computation intensive applications in wireless sensor networks because it offers high performance with low energy profile, easy integration of multiple types of sensors, and flexibility. This type of sensing platforms will therefore be suitable for the distributed data analysis and decision-making capabilities the emerging IoT applications require.

## 1. Introduction

The Internet of Things (IoT) is based on the vision of connecting physical things or objects to the Internet to enable access to distributed sensor data and to provide the ability to monitor and control the physical world from a distance [1]. It was envisaged that around 200 billion sensor devices would be interconnected via the IoT by 2020 [2], providing a tremendous capability to monitor and control physical phenomena within the environment [3].

The underlying technology that enables the realization of IoT is the Wireless Sensor Network (WSN) [4]. The WSN is a class of distributed systems that provide a bridge between the electronic/cyber and the physical worlds [5], and hence, will play a key role in the realisation of the IoT. Today, WSNs are capable of gathering physical environmental information, processing it, and transmitting the processed information to remote servers or base stations [6]. Traditionally, sensor nodes have been mostly comprised of scalar sensors capable of measuring physical phenomenon such as temperature, pressure, light intensity, humidity, etc. These traditional applications do not send a huge amount of data over the network and the information processing that needs to be performed on the sensor node is low. Such processing requirement can be handled by microcontrollers with standard processing capabilities. Examples of this type of processors are the ATmega16U2 in the Arduino Uno board [7], the ATmega128L in the Mica2, MicaZ motes, or the MSP430 in the Waspmote [8].

The IoT is increasingly being adopted in diverse fields, not only for monitoring, alert generation, and remote control, but also to achieve advanced control through automatic detection of patterns and prediction of future events (e.g., failure of a machine) [9], potentially delivering significant convenience, efficiency gains, and cost savings. Such advanced levels of control rely on intelligent analysis of data obtained from a large number and types of sensors. The approaches currently used predominantly rely on centralised data analysis in a cloud server, which requires all sensed data to be transmitted to the server. This involves significant energy dissipation at the sensor node, large communication time, bandwidth, and cloud storage capacity. Further, the timely preprocessing of data in central servers may be infeasible for real-time control. To address these issues and achieve real-time forecasts, researchers are focusing on intelligent techniques to reduce data and new distributed data analysis methods executed directly at the sensor nodes themselves [3]. This would necessitate a new generation of sensor nodes capable of supporting the required local signal processing and data analysis capability. For example, in the case of Wireless Multimedia Sensor Networks (WMSN) [10], the sensor nodes are required to possess powerful processing capability to be able to deal with the complexity of processing large volumes of multimedia data. The sensor nodes are still required to maintain a low power consumption profile, which allows the nodes to run on battery for long periods of time or rely on power harvested from the environment [11]. One way to deal with the high processing demand in the sensor nodes with relatively low energy consumption is to use tailored processing elements programmed on FPGAs rather than using off-the-shelf microcontrollers.

Several FPGA-based sensor platforms have been introduced [10,12,13,14,15,16]. Using FPGA can add powerful processing capability to a sensor node with reduced energy consumption, provided the amount of logic circuit used is optimized for the desired functionality and is activated only when required. Recent results show that by taking advantage of hardware acceleration together with power-aware designs and power management techniques, it is possible to obtain energy efficient solutions that are suitable for high-performance IoT applications [10,16,17]. Besides the advantage of high processing power, utilizing FPGA in the sensor nodes can provide flexibility by enabling the nodes to adapt to varying functional and performance needs of the applications.

The wireless sensor nodes reported in [10,17] are built on COTS FPGA boards. These FPGA boards are designed for multipurpose applications, and hence, there are redundant components. These components consume energy even when they are not needed. In this work, a new FPGA-based sensor node design is presented. For the majority of the FPGA-based sensor platforms mentioned above, the full details of the hardware architectural design, the interfaces, memory, IO and communication designs, and FPGA programming approach are not available. The work presented in this paper provides as many design details as are relevant to help readers comprehend the design and implementation from a very practical point of view. Most importantly, unlike the existing FPGA-based sensor node platforms, the platform proposed in this paper does not require an external microcontroller or softcore processor for any form of reconfiguration, thereby significantly reducing hardware and power consumption. The FPGA has been programmed with processing elements optimized for scalar sensing applications (e.g., temperature) as well as for processing and transmitting multimedia data (images). We present a diverse range of practical results on the power consumption of the FPGA node. The results demonstrate the efficacy of the FPGA-based sensor node containing tailored hardware processing elements for various applications including image communication over WMSN with acceptable power consumption.

## 2. Review of WSN Platforms

### 2.1. Microcontroller Versus FPGA Approach

Wireless Sensor Networks (WSN), the technology that underpins the IoT, have evolved dramatically with many new applications emerging in the last few years. So far, the most common applications were related to environmental care, agriculture, or hazardous environment monitoring [11]. Normally, these applications share some common features such as low data rate, unrestrictive latency, and a small number of nodes, among others. For these reasons, the processing requirement per node is low; therefore, low-profile processors can be used. These small processors support adequate processing capabilities for the above applications while offering ultra-low power consumption. The MSP430 microcontroller from Texas Instruments used in the TelosB [8] platform or the ATmega1281 microcontroller used by Libelium in the Waspmote [8] are examples of such low-profile processors.

However, low data rate and low power should not be a limitation of WSN and IoT applications. As technology continues to evolve, new applications that require more powerful processing units continue to emerge. For example, multimedia applications such as video and image processing have been investigated in the WSN field. These applications require much more powerful processors to perform compression or encryption of multimedia data. Consequently, higher processing speed and larger memory are needed, which also means that the issue of consequent higher power consumption needs to be addressed. These have been the major impediments for making multimedia applications of WSN or IoT a reality.

To achieve multimedia processing capability in WSN/IoT nodes, the use of more powerful microcontrollers can be one possible solution [18]. However, because microcontrollers are designed for generic applications, they are not optimized for processing intensive applications such as image processing. This solution would lead to very high computing time and high energy consumption. Smart energy management strategies can be used to reduce energy consumption, where the sensor node is kept *awake* only when required to perform a task; otherwise, it is put to *power down* or *sleep* mode [10,11,19]. However, as the microcontrollers need a large amount of time to finish complicated tasks such as image processing, they will need to be *awake* for long durations. This leads to high energy consumption, even with a smart energy management strategy.

The best way to reduce the *awake* time for complex processing applications is using dedicated hardware such as FPGAs or ASICs. FPGAs and ASICs share the capability of doing tasks in parallel. This can greatly decrease the computing time, so the node can be in the *awake* mode for very short period of time and stay in the *sleep* mode for longer periods of time. However, the use of ASICs in WSN/IoT nodes is not always feasible due to the lack of flexibility and the huge design time and cost. On the contrary, FPGAs are presented as an attractive solution for high-performance WSN/IoT applications. One major concern with using FPGAs for WSN/IoT nodes is that most off-the-shelf FPGA platforms are not specifically built for such applications [20]. This leads to redundant components on the available off-the-shelf FPGA development platforms, which will consume additional energy. For FPGA platforms to be suitable for IoT applications, their energy consumption, cost, and size need to be comparable with the microcontroller-based platforms, if not less.

In the next subsection, we summarize the review of notable sensor node platforms. Some of the parameters stated in the review will be used to compare the design of the FPGA-based sensing platform introduced in this paper.

### 2.2. Summary of Sensor Node Platforms

We classify wireless sensing platforms into three main categories, namely, (i) simple and low-power microcontroller-based platforms, (ii) powerful microcontroller-based platforms, and (iii) FPGA-based platforms. The sensor nodes in the first category are limited by their capabilities in terms of processing power, memory, and communication. However, they are typically characterized by low power consumption profiles. Platforms in this category includes the Mica family, IRIS, TelosB, etc., as shown in Table 1.

The second category includes powerful microcontroller-based platforms. The sensor nodes in this category use high-end processors and, therefore, have more processing capability than the nodes introduced in Table 1. However, these platforms consume a lot more power than those in Category 1 and usually tend to be more expensive. Platforms in this category include the Stargate, Arduino Yun, Beagle Bone family, Intel Galileo, and Raspberry Pi family, as shown in Table 2.

The third category includes FPGA-based platforms. Several FPGA-based sensor node platforms have been proposed in [15,20,24,25,26,27,28,29]. Valverde, Otero, Lopez, Portilla, De La Torre, and Riesgo [15] presented a high-performance sensor node architecture based on partial reconfiguration of an FPGA. It uses a Virtual Architecture to define the size and position of the reconfigurable components and the static components of the FPGA. The sensor node in [15] used a MicroBlaze *softcore* processor as the system controller and a Processor Local Bus (PLB) based on a System-on-Chip (SoC) approach to allow communication among various modules. A compression algorithm is used to reduce the size of the configuration *bitstream* and, hence, the programming time as well as power consumption. In another work by the same authors [30], a detailed explanation of the partial reconfiguration process is presented. A few other architectures based around partial reconfiguration also make use of softcore processors to control the reconfiguration process, for example [31], to implement secure remote reconfiguration. Most of the FPGA-based sensor platforms reported utilize commercial-off-the-shelf (COTS) FPGA development boards; hence, they have hardware redundancies. Further, thorough analysis of the power consumption of these platforms for WSN applications is not available. A list of FPGA-based sensor platforms is shown in Table 3. All of these platforms except [15] have reported high active power consumptions ranging from 1 W to 5 W. With a reported power consumption of 462 mW, the platform in [15] seems to be suitable for operating as a battery powered wireless sensor node. However, the 462-mW power consumption reported in [15] does not include the standby power consumed by the transceiver or the power consumed for wireless data communication.

## 3. Design Considerations

The FPGA-based sensor node introduced in this paper was conceived as one that would support a wide range of sensing and processing tasks. It was to be designed and developed as a single-board wireless sensor node with the capacity to easily plug in suitable sensing modules. So, the sensor node needed to have flexible IO, enabling the use of different sensors, transceivers, and other peripherals. Furthermore, it was necessary to have some *scalability* so that efficient operation for a wide range of sensing tasks could be achieved. This included the ability to sense scalar quantities such as temperature and humidity as well as sensing large data items such as images. The latter warranted the development of a high-performance sensor node capable of processing images captured by an on-board camera. At the same time, it was imperative that power consumption be kept as low as possible so that the batteries did not deplete energy quickly and the node could have a reasonably long lifetime in order to support distributed and autonomous operation in a wireless sensor network.

To cater for different sensing and associated processing tasks, Field-Programmable Gate Array (FPGA) was chosen as the central processing element so that the hardware functions required for various sensing tasks could be reprogrammed into the node as required. A longer-term goal was for the node to have the capability to store multiple FPGA configuration *bitstreams* so that the device could be reconfigured *in-system* to support changing sensing and processing requirements. Hence, there was the need to carefully consider the size of the FPGA configuration file; the number of configuration files to be stored; and consequently, the on-board flash memory size required. Another important design consideration was the interfaces to be used to supply power and to program the sensor node. The aim was to use a single USB interface for both purposes.

## 4. The Proposed Sensor Node

**Figure 1:**
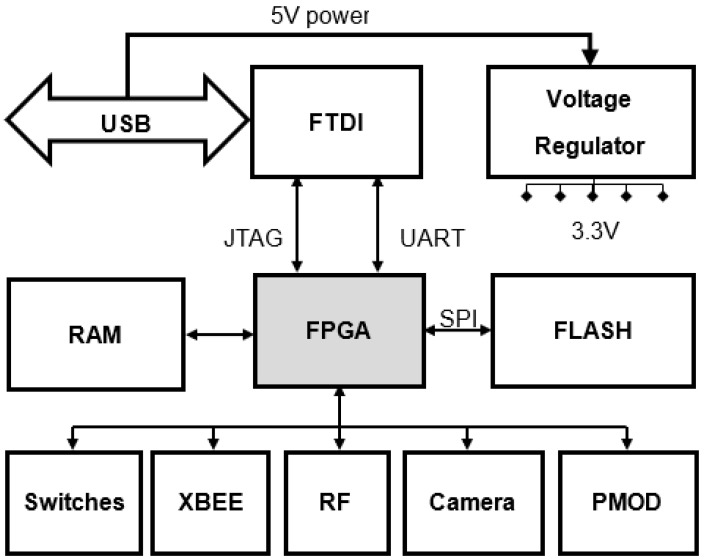
Block diagram of the Wireless Sensor Node.

Figure 1 shows a block diagram of the proposed sensor node. As stated above, an FPGA was selected over a microcontroller for the central processing unit to provide the required computational power while maintaining comparatively low power requirements and provide flexibility to easily reconfigure the hardware functions. The Spartan 6 family of FPGAs from Xilinx was identified as a potential contender due to their low power design and features [35]. In-house printed circuit board (PCB) mounting capabilities did not support packages that came with the Ball Grid Array (BGA). For this reason and considering availability and cost, the XC6SLX9 FPGA device in the TQG144 quad-flat-package was selected. This provided 102 user I/O and 1430 slices (each slice contains four 6-input LUTs and eight flip-flops) [36]. A 100-MHz oscillator was used to provide the system clock.

**Figure 2:**
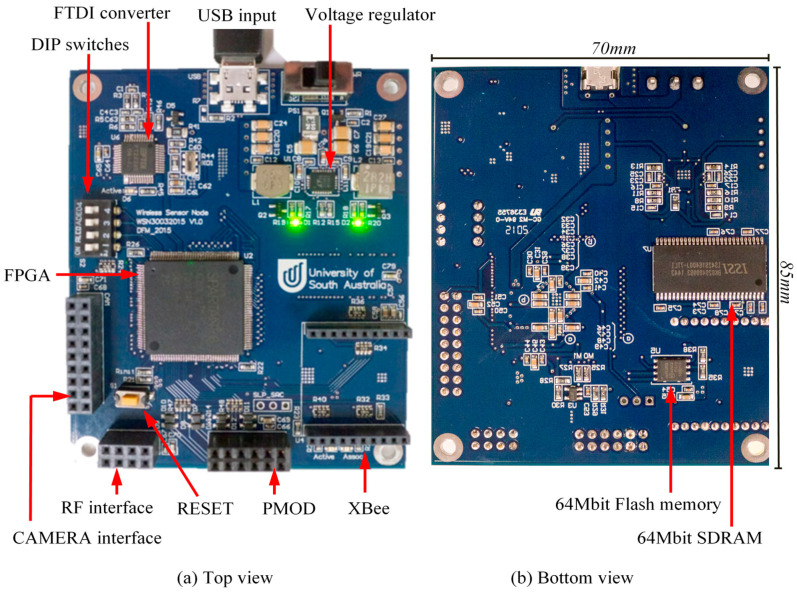
FPGA-based Wireless Sensor Node—dimensions 70 mm × 85 mm.

A photograph of the base sensor node (all peripheral modules removed) is shown in Figure 2.

### 4.1. Memory and IO

To store the *bitstreams* required to configure the FPGA, a 64 Mbit Flash memory module is connected to the FPGA via serial peripheral interface (SPI). A 64-Mbit SDRAM is also provided to enable more complex applications such as image processing. The Flash memory and the SDRAM are mounted on the bottom of the 6-layer PCB (Figure 2) in order to conserve space.

The various IO headers supported by the sensor node are labelled in Figure 2. The *Reset* button at the bottom-left corner of Figure 2a allows manual reconfiguration of the FPGA using the *bitstream* stored in the Flash memory. The four DIP switches on the left-hand side are used for user input. Headers are included for connecting cameras (far left of Figure 2a) and PMOD devices (bottom of Figure 2a). PMOD is an interface standard defined by Digilent [27]. This interface was selected because over 60 I/O and sensor boards are available from Digilent, ranging from GPS receivers and Wi-Fi modules to motor drivers and temperature sensors [37]. Two other headers shown in Figure 2, namely, the RF Transceiver and XBee Pro, are described later.

### 4.2. Programming Interface and Power Supply

A dual-channel *USB-to-serial* converter IC, the FTDI FT2232D [38], was used to achieve USB connectivity. The first channel is used for programming the FPGA via JTAG. The second channel can be utilized in user designs as a UART interface connected to the FPGA, thereby enabling easy communication between the FPGA and external devices. Compared to other more advanced devices [39], the FT2232D achieved the required function of providing a JTAG channel for programming the FPGA with less complex circuitry.

Power is supplied through the USB connection from either a USB power source or a battery pack containing 4 standard AA batteries. A switch-mode buck regulator was used to regulate the input voltage to the levels required by the devices on the node. All components on the sensor node are supplied with 3.3 V including the headers. The FPGA is additionally supplied with 1.2 V for the internal circuitry. The USB input, the FTDI USB converter and the voltage regulator can be seen on the top part of Figure 2a.

### 4.3. Wireless Communication

The sensor node has been designed with headers to allow the connection of two different wireless communication transceivers. The header at the bottom-right corner of Figure 2a is for housing an XBee Pro S2B module to implement a ZigBee network [40]. This provides off-the-shelf RF transmission in the ISM 2.4 GHz band for data rates of up to 35 kbps. The module implements a high-level application layer and can be operated in *API mode*, where the user interacts with the raw packets, or in *AT mode*, where the module acts as a transparent serial UART connection. A wireless network of XBee modules can be automatically configured with the ability to support both star and mesh topologies. The XBee modules in the network can receive broadcast messages. Each module can also be addressed individually. The XBee modules feature a *sleep* mode whereby they enter a low-power state to conserve energy.

A second header is available to support a standard RF module (bottom-left of Figure 2a). This is to allow easy interfacing of an 802.15.4 transceiver and to facilitate the development of further energy efficient, lightweight application layer protocols based on 802.15.4. In the work presented in this paper, standard RF transceivers were not utilized. Only XBee Pro S2B modules were used to set up a wireless network. These were used in the *AT mode*, which enabled a straightforward network configuration.

## 5. FPGA Programming

Once a user design has been successfully synthesized on a PC and a configuration *bitstream* is generated, it can be used to program the FPGA via the USB connection. The FTDI USB converter produces the JTAG signals required to interact with the FPGA. However, the configuration memory of the Spartan 6 FPGA is volatile. This means that once power to the FPGA is lost, the memory is erased. Consequently, it is standard practice to install external, nonvolatile memory alongside the FPGA. Upon receiving power, the FPGA will then automatically load a configuration *bitstream* stored on the nonvolatile memory. Among the five configuration modes supported by the Spartan 6 FPGA family [41], the *Master Serial/SPI* mode is used in the proposed sensor node to load a *bitstream* from the Flash memory to the FPGA.

### 5.1. Manual Reconfiguration

In order to generate a valid configuration file for the sensor node, Xilinx ISE was used to synthesize a *bitstream* for the FPGA from VHDL code. Once the *bitstream* is available, this can be used to directly configure the FPGA using the Xilinx iMPACT tool by utilizing the Xilinx Virtual Cable (XVC) function in conjunction with the *Playtag* Python tool [42] to ensure that the JTAG chain is discovered by iMPACT. This has the limitation of writing the configuration directly to the FPGA’s volatile memory, meaning the configuration is lost when power is removed.

The iMPACT software was apparently unable to access the Flash memory on the sensor node. However, another FPGA programming software was identified that could achieve this. It is an open source command line tool called XC3SPROG [43]. It is able to program a range of FPGAs and is available for Windows, Linux, and Mac OS X platforms. As the Flash memory is not connected to the FTDI USB converter, the FPGA is first configured with a special *bitstream* that allows the FPGA to act as a JTAG to SPI converter. XC3SPROG is then able to store the *bitstream* in any specified location on the flash memory.

When the FPGA is first powered, it automatically loads a configuration from address 0x00000000 of the Flash memory. This may be a valid configuration or a short header that redirects the FPGA to a different memory address to load the configuration from.

### 5.2. Reconfiguration Using Multiboot

**Figure 3:**
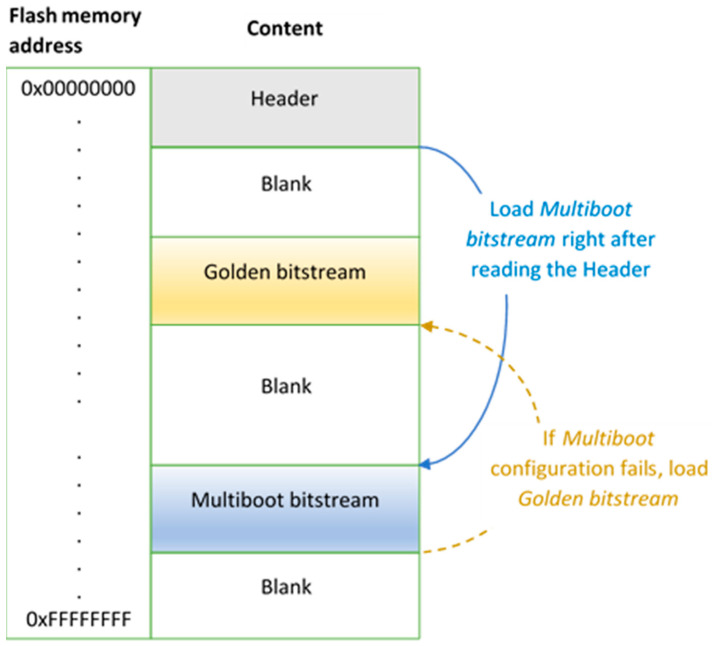
*Multiboot* configuration process [41].

The Spartan 6 family of FPGAs can also be programmed using a feature called *Multiboot*. It allows the FPGA to selectively reload and reprogram its *bitstream* from the external Flash memory [41]. *Multiboot* operations can be controlled by interacting with the configuration logic of the FPGA using the internal configuration access port (ICAP). The ICAP can be accessed from a user’s configuration by including the ICAP_SPARTAN6 primitive into the configuration design. There are four registers within the configuration logic. These registers are used to store the memory address of the *bitstreams* that are to be loaded to configure the FPGA. These registers can be set through the ICAP or through a short header *bitstream* located at address 0x00000000 of the external memory. The GENERAL1 and GENERAL2 registers are used to store the address of the *Multiboot bitstream* that the user wishes to load. The GENERAL3 and GENERAL4 registers are used to store a fallback address where a “Golden” *bitstream* is stored, as shown in Figure 3. This *Golden bitstream* is known to be safe and is usually located in a protected area of the flash memory. Once the *bitstream* addresses have been set in the registers, the ICAP is used to issue an IPROG command [41]. This has an effect similar to manually resetting the FPGA except the *Multiboot logic* is not affected, allowing the contents of the registers to be used to set the address from which to load the *bitstream*. Further details of the Spartan 6 FPGA configuration process can be found in [41].

In order to trigger the FPGA to reload its configuration from a specific memory location on the SPI flash, a finite state machine was implemented to interact with the ICAP. Initially, when a “LOAD” command is received over the network, the FSM will receive three additional bytes specifying the address to load the configuration from. Once this address has been received, the FSM writes the address into the GENERAL1 and GENERAL2 registers using the ICAP. Finally, an IPROG command is issued causing the FPGA to reconfigure from the specified Flash memory address. One important aspect to note here is that with the “LOAD” command received over the network, it is possible to remotely reconfigure the sensor node from *bitstreams* prestored in specific Flash memory locations. This makes the proposed sensor node very attractive compared with existing FPGA-based sensor platforms because, unlike the existing platforms, it does not require a separate microcontroller or softcore processor to manage the reconfiguration process.

Having a copy of the reconfiguration architecture as the *Golden bitstream* in a protected area of the flash allows for easy recovery of the sensor node if a configuration error is encountered. If an incorrect address is provided or the *bitstream* at the specified address is corrupted, the FPGA will automatically load the *Golden bitstream*, which is expected to be a safe version of the reconfiguration architecture. A new address can then be provided or a new *bitstream*, which is uploaded and written to the Flash memory.

## 6. Wireless Networking Set up and Applications

A network was set up with 9 of the developed FPGA-based sensor nodes. Additionally, 9 sensor nodes based around a traditional microcontroller-based platform (Arduino) [7] were added to provide diversity and allow experimentation in a heterogeneous network consisting of at least two types of nodes. The same XBee transceivers were used on both types of nodes to enable seamless communication in a mesh network containing a total of 18 endpoints.

**Figure 4:**
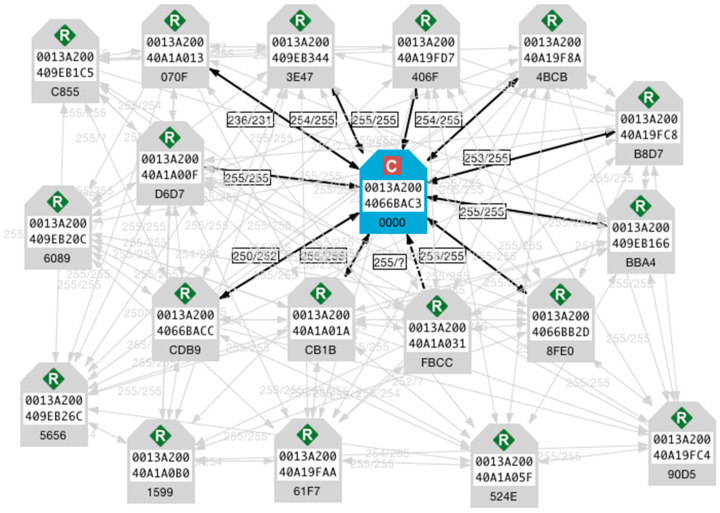
Schematic view of the ZigBee mesh network with 18 endpoints.

Figure 4 shows a schematic view of the network, where a central coordinating station called *gateway* provides access to the *Internet*. The blue node in the middle (marked ‘C’) indicates the coordinator XBee device connected to the *gateway*. The dark arrows indicate the direct connections to the coordinator and the light arrows show the mesh connections between the remaining nodes.

A commercial off-the-shelf (COTS) platform called ZedBoard [44] was used as the *gateway*, with much higher computational power than the ordinary sensor nodes. A modified version of the Linaro operating system [45] was installed on the *gateway*. The *gateway* was connected to the *Internet* via a Wi-Fi network. Software programs were developed in C++ and Python to run on the *gateway*, which would capture the data received by the coordinator module and upload it to the Cloud to allow remote access. The Dropbox API [46] was used as the Cloud service.

The sensor nodes in the network (Figure 4) were programmed with digital logic to support various sensing and actuation applications, thereby forming a network of heterogeneous sensing nodes. These include sensing of low-volume scalar parameters such as temperature, humidity, and motion as well as large-volume data such as images. The actuation system implemented on the sensor node included simple logic to operate everyday appliances (light, fan, etc.) via power switches controlled using optical isolators interfaced with the sensor node.

### 6.1. Temperature Sensing

Many COTS sensors are equipped with UART, I2C, or SPI interfaces. Using FPGA as a sensor node provides the flexibility of programming the necessary logic within the FPGA to deal with various sensing parameters and multiple types of sensor interfaces. The *FlexiS* was evaluated with both I2C and SPI temperature sensors and the required resources as well as power consumption were analysed for both cases. A PmodTMP3 sensor module with I2C interface and a PmodGYRO module with SPI interface were used for temperature sensing. Both of these modules are available from Digilent [37].

**Figure 5:**
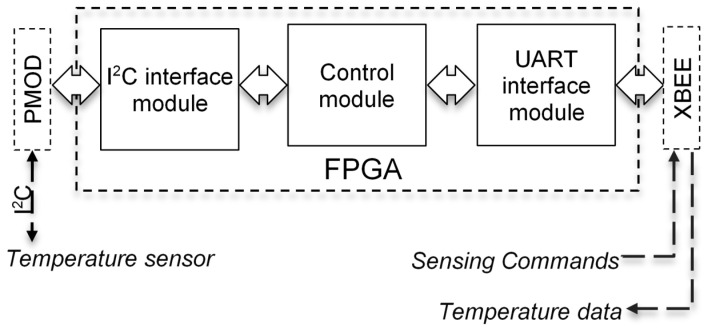
Block diagram of temperature-sensing logic.

Figure 5 shows the block diagram of the digital logic system developed for temperature sensing with I2C interface. In Figure 5, a PmodTMP3 module from Digilent [37] was attached to the PMOD port of the sensor node. A temperature sensor that has a SPI interface instead, such as the PmodGYRO module from Digilent [37], can also be connected to the PMOD port in Figure 5. Of course, this will require the relevant SPI logic to be programmed into the FPGA. The PmodGYRO module can provide 3-axis gyroscope data as well as temperature.

**Figure 6:**
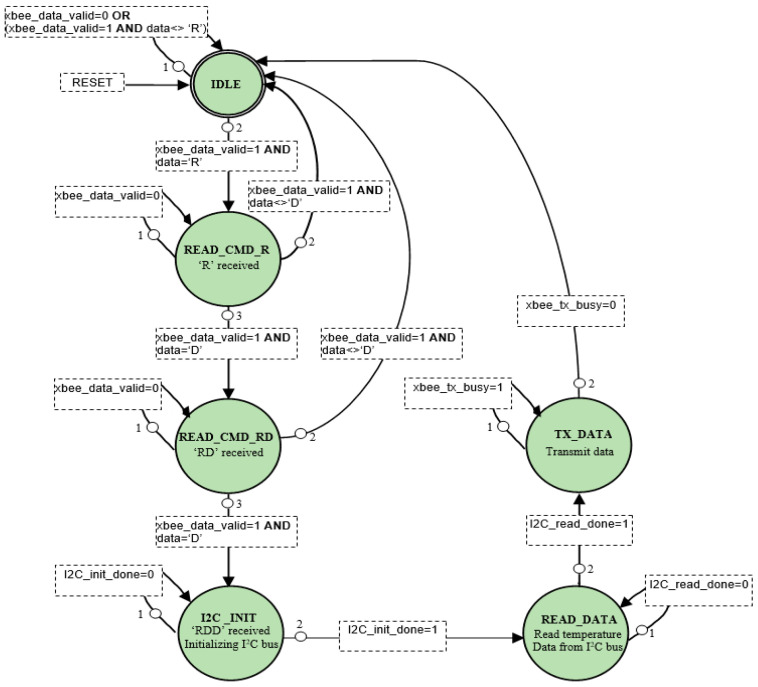
State diagram of I2C temperature-sensing logic.

The Control Module shown in Figure 5 is a state machine designed to accomplish the following tasks: (1) send necessary commands and initialize the temperature sensor; (2) receive sensed data from the sensor via the relevant interface (I2C or SPI); (3) make all sensed data available at its outputs so that the data can be transmitted over the network. The state machine developed to implement the I2C temperature sensing system is shown in Figure 6. It comprises of 6 states: IDLE, READ_CMD_R, READ_CMD_RD, I2C_INIT, READ_DATA, and TX_DATA.

In the IDLE state, the control block waits for the initial command from the network, which is the ASCII character “R”. When “R” is received (via the UART interface module), the state moves to READ_CMD_R and waits for the second character of the command, which is “D”. When ‘D’ is received, the state moves to READ_CMD_RD. Then, it waits for a second ‘D’. After the correct sequence (‘RDD’) is finally received, it moves to the I2C_INIT state and starts initializing the I2C bus.

When the I2C initialization is complete, the state moves to READ_DATA, where the temperature data is read from the PmodTMP3 module. When the reading process is complete, the state moves to TX_DATA, where the sensed data is sent via the UART interface to the XBEE module and then to the base station. After data transmission is complete, the state returns to IDLE and waits for the next command.

The power consumption and logic resources required for the I2C temperature-sensing system described above (Figure 6) are given in Table 4. Note that Table 4 shows the above information for each of the individual logic blocks designed as part of the overall state machine.

### 6.2. Simple Actuation and PWM Fan Control

**Figure 7:**
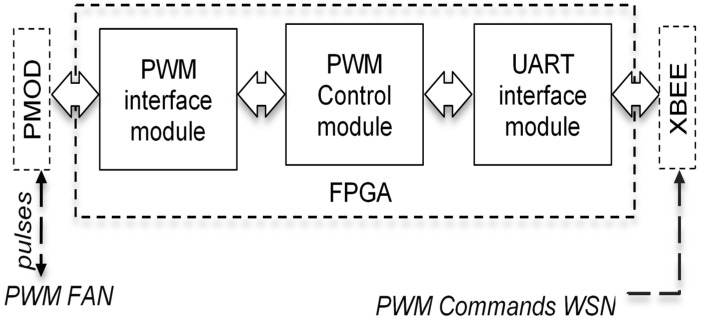
Block diagram of PWM fan control logic.

**Figure 8:**
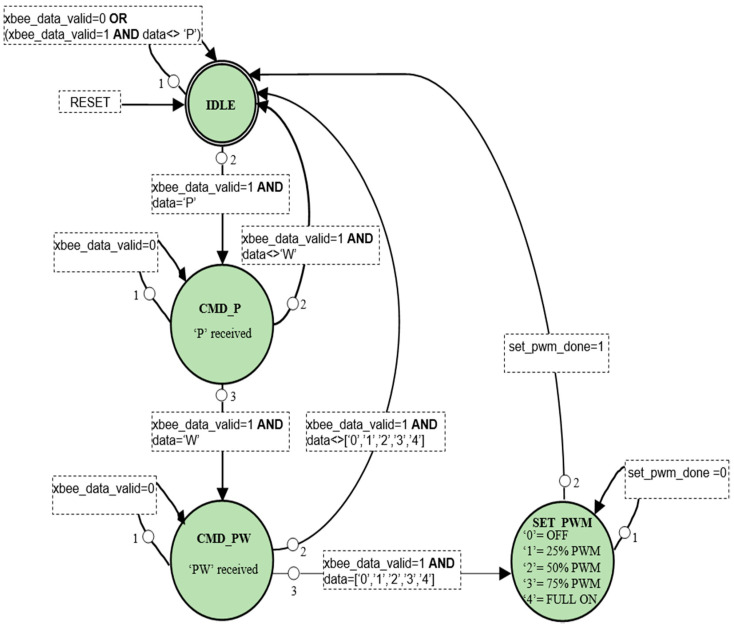
State diagram of PWM control logic.

Figure 7 shows the block diagram of the scheme developed for the PWM fan control application. In general, the state machine for PWM fan control is similar to that for temperature sensing presented in the previous section. It comprises of 4 states: IDLE, CMD_P, CMD_PW, and SET_PWM. In IDLE state, the control block waits for the command from the network, which is the ASCII sequence of “PW0” to “PW4”, where “PW0” is to turn *off* the PWM fan and “PW4” is to turn the PWM fan full *on*. “PW1”, “PW2”, and “PW3” commands are used to control the PWM fan speed at 25%, 50%, and 75%, respectively. The state machine developed for the PWM fan control is shown in Figure 8.

Note that the state machine given in Figure 8 needs only slight adjustments to implement AC switch control functions. For example, “PW1” and “PW2” can be used to turn ON or OFF one AC switch, whereas “PW3” and “PW4” can be used to turn ON or OFF a second AC switch. Comparison of power consumption and logic resources required for the PWM fan control application and I2C temperature sensing application are shown in Table 5. The maximum frequency of operation (*F_max_*) achievable by the two controllers are also shown in Table 5.

## 7. Experiments and Results

Both scalar quantities and large data items (images) were transacted over the wireless network that was set up (Figure 4) and described in Section 6. Since energy is a concern for battery powered nodes, in this section, we present a diverse range of practical results on power consumption for the scalar applications presented in Section 6 as well as for image processing applications [10,17].

### 7.1. Test Applications

The proposed sensor node has been used for a variety of applications, namely, sensing of temperature and humidity, simple actuation (ON/OFF control), and PWM fan control. In addition, the node was interfaced with image sensors (CMOS cameras) and the captured images were communicated over the wireless sensor network. To reduce energy consumption, efficient object extraction techniques [17] were implemented on the FPGA to only send updated objects in the image frame rather than the full image. To reduce energy consumption when full images need to be transmitted, an efficient image compression architecture [26] was also implemented on the proposed sensor node.

### 7.2. Real-Time State Transitions

**Figure 9:**
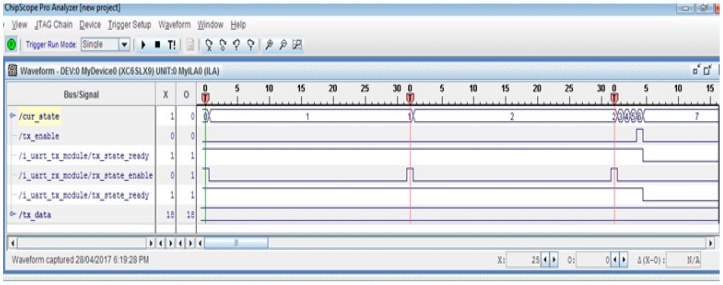
Real-time plot of state transitions during temperature sensing using ChipScope tool.

The real-time state transitions of the FSMs implemented on the FPGA were monitored using the Xilinx ChipScope tool. The captured waveforms for the temperature-sensing application are shown in Figure 9. It shows all the states of the FSM (*cur_state*) during temperature sensing and during transmission of the sensed data. The transmitted temperature data are also shown (*tx_data*).

### 7.3. Graphical User Interface

**Figure 10:**
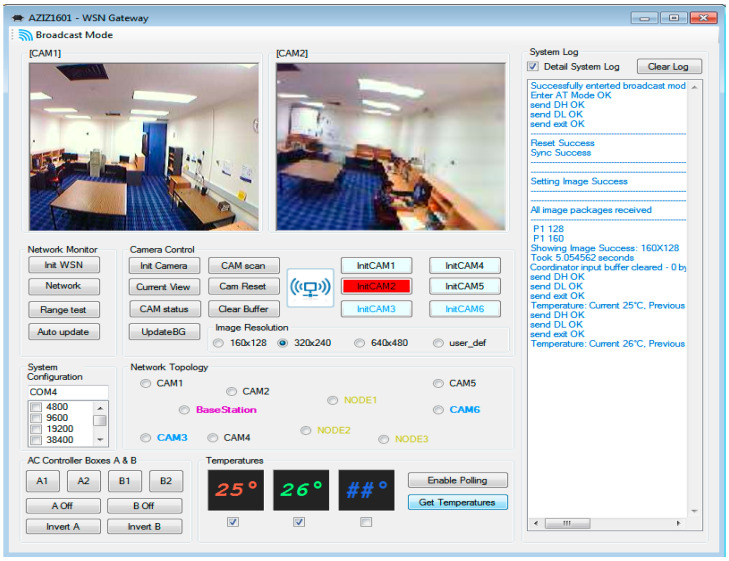
Graphical User Interface (GUI) at the base-station.

Figure 10 shows the GUI developed for various sensing and control applications as well as image-processing applications. Compressed images received from two different camera nodes over the network are depicted within Figure 10. The two images depicted have two different resolutions that are selected from within the GUI. The control switches and sensed temperatures can be seen at the bottom part of Figure 10.

### 7.4. Power Consumption

For all experiments, power consumed by the sensor node was determined by measuring the current flowing through the power supply rail. In order to assist with collecting accurate power measurements, one of the pins in the PMOD header of the sensor node was used as a trigger for the digital storage oscilloscope (DSO). As soon as the sensor node leaves the IDLE state, this pin is set to *high*, causing the DSO to trigger and record power data during the *receive* and *send* states. Additionally, in order to measure the power consumed by the XBee module in *sleep* mode, a configuration was developed to assert the XBee *sleep* pin. This required virtually no logic, resulting in a “minimal configuration”.

**Figure 11:**
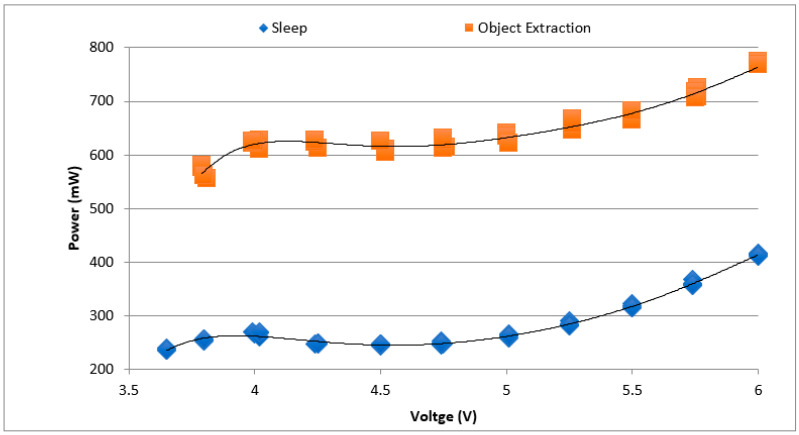
Power consumption profile of the sensor node for object extraction in *data processing* mode and *sleep* mode.

The power consumed by the sensor node was measured with an object extraction architecture programmed into the FPGA. Figure 11 shows the experimental results on the power consumed by the sensor node at various supply voltages. At 5 V supply, the power consumed by the object extraction architecture is 620 mW. When the FPGA is put to *sleep* mode, the power consumption reduces significantly, consuming only 250 mW at 5 V.

**Figure 12:**
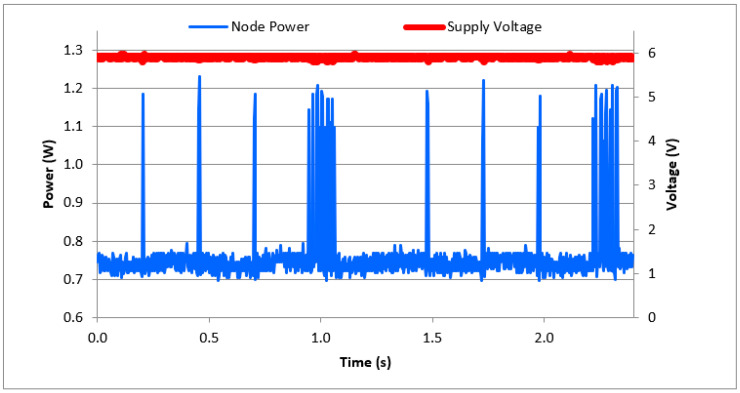
Power consumption of the sensor node during image capture, processing, and transmission operations.

**Figure 13:**
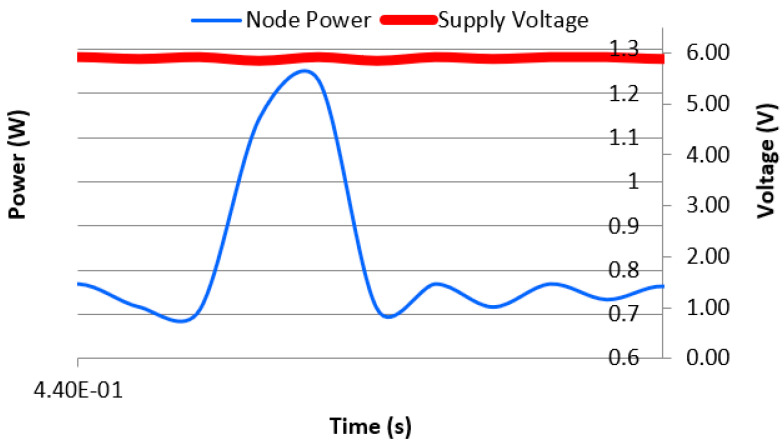
Zoomed-in view of the power consumption from Figure 12.

We also programmed both the object extraction and the image compression architectures into the FPGA. For simplicity, we refer to these combined operations as image processing. Figure 12 shows a partial snapshot of the real-time power consumption of the sensor node during the combined object extraction and compression operations (~0.75 W), and during data transmission (~1.2 W). A zoomed-in view of one transmission transaction is shown in Figure 13. These measurements were obtained when the node supply voltage was set to 6 V DC.

**Figure 14:**
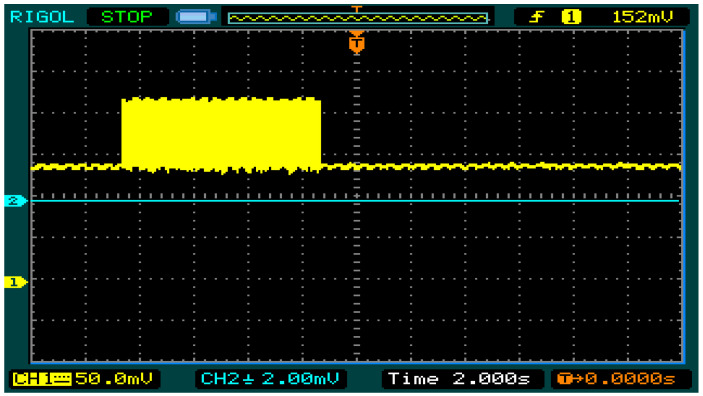
Power consumption for the image processing and transmission cycle.

For a more comprehensive view of the performance during the combined image-processing operation, in Figure 14, we have captured the power consumption for an entire image processing and transmission cycle. In this example, the time taken for the entire processing and transmission operation is approximately 7 s. This is likely to vary depending on the nature and size of the extracted object (image) as well as the network configuration and prevailing network conditions (noise levels, number of hops, etc.).

As stated previously, a few simple applications such as temperature monitoring and switch/fan control as well as more complex applications such as image compression and object extraction have been implemented on the FPGA. Power consumption for each application has been measured and is shown in Table 6.

Table 6 shows that even with the most complicated data-processing (image-processing) application, the proposed sensor platform consumes only 713.6 mW of power, which is the same as the power consumption of the Stargate node reported in Table 2. This power consumption is less than half of all the other off-the-shelf platforms presented in Table 2. In addition, this power consumption is less than all the FPGA-based sensor nodes listed in Table 3, except the one reported in [15]. However, the authors of [15] did not report the standby power consumption of the wireless transceiver or the power consumed for wireless data communication. For low data rate (scalar) processing applications such as those reported in Table 6, *FlexiS* consumes almost the same or less power. This demonstrates that the proposed platform is competitive for a variety of applications that require complicated data processing but have limited energy resources. In fact, it is more attractive than the majority of the platforms presented in Table 2 and Table 3.

One additional benefit of using such a platform as *FlexiS* is the processing time. As reported in [17], for a complex data processing task, the FPGA processing node may consume less power by finishing the task quickly and then going back to *sleep* mode.

## 8. Conclusions

This paper has presented *FlexiS*, a high-performance, flexible, embedded sensor node platform based on FPGA. Unlike exiting literature on FPGA-based sensor nodes, this paper has provided the hardware architecture and other design details as well as the implementation of finite state machines. It has been successfully applied in a range of applications dealing with both scalar and high-volume data. Test results demonstrate that *FlexiS* is highly suitable for data and computation-intensive applications in wireless sensor networks because it offers high performance with a competitive energy profile, flexibility, and the ability to easily connect multiple types of sensors. Clearly, this type of sensing platform can be used to deliver the distributed processing and data analysis capability required by emerging IoT applications. Being able to reprogram the hardware functions within the FPGA means that the proposed sensor node can be easily adapted to various IoT applications. It can also be easily adapted *in-system* when the sensing environment changes, for example, if the sampling rate needs to be adjusted. The proposed sensor node platform can help implement a range of seamlessly connected and adaptable sensing devices, which in turn, is likely to harness new capabilities in future IoT.

## Figures and Tables

**Table 1:** Microcontroller-based low-power platforms.

Platform	Processor	Memory	Transceiver	Active Power (mW)	Active Power + RF (mW)	Price
MicaZ [21]	ATMega128L	128 KB program memory, 4 KB SRAM	CC1000	43.2	71	$92
IRIS [22]	ATmega1281	128 KB program memory, 8 KB SRAM	Atmel AT86RF230	28.8	61.2	$29
TelosB [23]	MSP430	60 KB program memory, 2 KB SRAM	CC2420	6.5	83	$79

**Table 2:** Complex microcontroller-based platforms.

Platform	Processor	Memory	Active Power	Price
Stargate	Intel PXA255 Processor	64 MB SDRAM, 32 MB Flash	600 mW	Unknown
Arduino Yun	MIPS32 24K and ATmega32U4	64 MB SDRAM, 16 MB Flash	1.4 W	$75
Beagle Bone Black	ARM Cortex-A8	512 MB SDRAM, 2 GB Flash	2.2 W	$55
Intel Galileo	Intel X1000	256 MB SDRAM, 8 MB Flash	3.5 W	$80
Raspberry Pi	ARM1176	256 MB SDRAM (Model B)	2 W	$35

**Table 3:** FPGA-based platforms.

Platform	FPGA	Memory	Active Power	Price
[15]	Spartan 6 XC6SLX150-2	256 Mbits	462 mW	Unknown
[32]	Artix-7 FPGA: XC7A35T-1CPG236C	1800 Kbits	5 W	$149
[31]	Virtex 5 XC5VFX70T	Unknown	Unknown	Unknown
[33]	Spartan-3E XC3S1600E	Unknown	2.85 W	Unknown
[34]	Spartan-3 XC3S2000	Unknown	1000 mW	Unknown

**Table 4:** Power consumption and FPGA resources used by I2C temperature sensing.

Block	Power (mW)	Logic Power (mW)	Signal Power (mW)	# FFs	# LUTs
Control block	0.19	0.08	0.11	14	22
uart_tx_module	0.15	0.10	0.05	18	22
uart_rx_module	0.13	0.08	0.06	18	22
uart_rate_control	0.10	0.06	0.04	7	9
i2c_master	0.77	0.42	0.34	39	68
Total	1.35	0.74	0.61	96	143

**Table 5:** Power consumption and logic resources for PWM fan control vs. temperature sensing.

Application	Programming Time	Total Power (mW)	Fmax	Resources	
				# FFs	# LUTs
Temperature monitoring	iMPACT: 10 s XC3Sprog: 2331 ms	1.35	292.483 MHz	96	143
PWM Control	iMPACT: 10 s XC3Sprog: 2330 ms	5.28	144.547 MHz	296	568

**Table 6:** Power consumption profile of the sensor node for different applications.

Setup	Processing Power at 5 V Supply (mW)
	Maximum
Temperature monitoring using SPI	473.55
Temperature monitoring using I2C	402.1
AC Switch control	432.8
PWM fan control	441.7
Image compression	675.2
Combined image compression and object extraction	713.6
